# Anatomical Engineering and 3D Printing for Surgery and Medical Devices: International Review and Future Exponential Innovations

**DOI:** 10.1155/2022/6797745

**Published:** 2022-03-24

**Authors:** José Cornejo, Jorge A. Cornejo-Aguilar, Mariela Vargas, Carlos G. Helguero, Rafhael Milanezi de Andrade, Sebastian Torres-Montoya, Javier Asensio-Salazar, Alvaro Rivero Calle, Jaime Martínez Santos, Aaron Damon, Alfredo Quiñones-Hinojosa, Miguel D. Quintero-Consuegra, Juan Pablo Umaña, Sebastian Gallo-Bernal, Manolo Briceño, Paolo Tripodi, Raul Sebastian, Paul Perales-Villarroel, Gabriel De la Cruz-Ku, Travis Mckenzie, Victor Sebastian Arruarana, Jiakai Ji, Laura Zuluaga, Daniela A. Haehn, Albit Paoli, Jordan C. Villa, Roxana Martinez, Cristians Gonzalez, Rafael J. Grossmann, Gabriel Escalona, Ilaria Cinelli, Thais Russomano

**Affiliations:** ^1^Facultad de Ingeniería, Universidad San Ignacio de Loyola, La Molina, Lima 15024, Peru; ^2^Department of Medicine and Biology & Department of Physics and Engineering, Bioastronautics and Space Mechatronics Research Group, Lima 15024, Peru; ^3^Department of Surgery, Northwest Hospital, Randallstown, MD, USA; ^4^Faculty of Health Sciences, Universidad de León, Spain; ^5^Escuela Superior Politécnica del Litoral, ESPOL, Ecuador; ^6^Robotics and Biomechanics Laboratory, Department of Mechanical Engineering, Universidade Federal do Espírito Santo, Brazil; ^7^Teodorescu Lab, University of California, Santa Cruz, CA, USA; ^8^Department of Oral and Maxillofacial Surgery, Hospital 12 de Octubre, Madrid, Spain; ^9^Department of Neurosurgery, Medical University of South Carolina, Charleston, SC, USA; ^10^Department of Neurosurgery, Mayo Clinic, FL, USA; ^11^Department of Neurosurgery, Cedars-Sinai Medical Center, Los Angeles, CA, USA; ^12^Cardiovascular Surgery, Instituto de Cardiología-Fundación Cardioinfantil, Universidad del Rosario, Bogotá DC, Colombia; ^13^Department of Radiology, Massachusetts General Hospital, Boston, MA, USA; ^14^Villamedic Group, Lima, Peru; ^15^Clínica Internacional, Lima, Peru; ^16^Hospital Universitari Sagrat Cor, Barcelona, Spain; ^17^Miami Cancer Research Center, FL, USA; ^18^Palmetto General Hospital, FL, USA; ^19^Universidad Científica del Sur, Lima, Peru; ^20^Department of Surgery, Mayo Clinic, Rochester, MN, USA; ^21^Obstetrics and Gynecology, Lincoln Medical and Mental Health Center, Bronx, NY, USA; ^22^Department of Urology, Fundación Santa Fe de Bogotá, Colombia; ^23^Department of Urology, Mayo Clinic, FL, USA; ^24^Howard University Hospital, Washington, DC, USA; ^25^Nouvel Hôpital Civil, Hôpitaux Universitaires de Strasbourg, Strasbourg, France; ^26^Institut of Image-Guided Surgery (IHU-Strasbourg), Strasbourg, France; ^27^Portsmouth Regional Hospital, NH, USA; ^28^Experimental Surgery and Simulation Center, Department of Digestive Surgery, Catholic University of Chile, Santiago, Chile; ^29^Aerospace Human Factors Association, Aerospace Medical Association, VA, USA; ^30^InnovaSpace, London, UK

## Abstract

Three-dimensional printing (3DP) has recently gained importance in the medical industry, especially in surgical specialties. It uses different techniques and materials based on patients' needs, which allows bioprofessionals to design and develop unique pieces using medical imaging provided by computed tomography (CT) and magnetic resonance imaging (MRI). Therefore, the Department of Biology and Medicine and the Department of Physics and Engineering, at the Bioastronautics and Space Mechatronics Research Group, have managed and supervised an international cooperation study, in order to present a general review of the innovative surgical applications, focused on anatomical systems, such as the nervous and craniofacial system, cardiovascular system, digestive system, genitourinary system, and musculoskeletal system. Finally, the integration with augmented, mixed, virtual reality is analyzed to show the advantages of personalized treatments, taking into account the improvements for preoperative, intraoperative planning, and medical training. Also, this article explores the creation of devices and tools for space surgery to get better outcomes under changing gravity conditions.

## 1. Introduction

Additive manufacturing (AM), also called three-dimensional printing (3DP), is a manufacturing process that has ramped its participation into industry as it offers unique characteristics in order to produce objects in a digital fabrication workflow. For several years, AM has paved its path into medical industry by creating useful and innovative solutions to daily common problems. These solutions are mainly group into three different categories: (1) AM used as presurgical tool, (2) AM used as intrasurgical tool, and (3) AM used as an implant or replacement [1]. Each one of these categories has posed and solved challenges for engineers and medical doctors, and, in this process, commercial solutions have been created and added to medical industry which is commonly used for design and construction of surgical mechatronic systems and anatomical training simulation procedures [2–8].

As a presurgical tool, AM has introduced a simple yet powerful tool for medical doctors and surgeons: physical 3D-printed models [9]. Created based on reverse-engineering of 3D medical data acquisition procedures, a virtual model with precise detail can be obtained [10]. Surgeons will use these models for procedure planning as they will have in their hands a replica of what they will find when they expose their patients during surgery [11]. Better outcomes, more reliable surgeries, costs saving, and shorter postoperatory procedures are among the benefits of using 3D-printed anatomical models [12].

As an intrasurgical tool, AM has helped doctors and engineers to create tools and devices that assists surgeons during medical procedures in the operating room (OR). One of the more developed devices is the 3D-printed surgical guide [13]. Guides are tools created by using a similar procedure as compared to anatomical models, however, they mimic an organ's complex surface to obtain a jig that will allow the surgeon to perform a cut, drill, or resection in a more precise and clean procedure. These guides have proved to more effective in resection as compared to typical free hand cutting techniques [13]. Other medical devices that use the potential of AM previously depicted in this article are also being developed, such as hearing aids, dental aligners, and frames for glasses [14, 15].

Finally, the application that is currently in the focus of researchers is how AM can be used as a functional implant and, in the future, as a fabrication technique for fully operative organs [16]. Being able to obtain anatomical models from medical images has open the discussion of whether these replicas might evolve into utile organs [9]. The scientific community has started to design and 3D-print scaffolds with intricate shapes that have proved to serve as a favorable medium to promote cellular activity and differentiation [17]. In the meantime, AM is currently being used to successfully create implants with complex shapes and topologies for orthopaedic and maxillofacial surgeries among others [18].

AM is not new, however, its introduction to the medical field was just in the last decade, and applications are still being developed with an increased demand from patients, hospitals, and insurance companies that have embraced Anatomical Engineering as a useful and high exponential growth field [19]. This article is the focus in surgery, which presents a clear and comprehensive view to some of the most interesting and promising applications of AM and the use of its potential to solve complex problems and, ultimately, increases the quality of life of patients [20, 21].

Since the beginning of the use of 3D printing in the medical field in the 1990s, there has been an exponential development in the different areas of surgery, which was initially to educate patients and surgeons; additionally, its use is being applied in the creation of new organs. Thereby, the following question was raised: what are the advances and the outcomes in the applications of 3D printing in surgery at the presurgical, intrasurgical, and postsurgical settings in each group of surgical subspecialties? The main objective of this work is to have a better knowledge of this developing technology and thus, with this highly specialized group of surgeons and engineers, be able to develop new 3D technologies and promote their concomitant use with high-resolution images: magnetic resolution image (MRI), computerized tomography (CT), and ultrasound (US) in order to have a high impact on the health of patients [22].

## 2. Additive Manufacturing Techniques

With the advent of CT and MRI, the medical field achieves the ability to visualize in-body accurate geometries without surgery intervention [23, 24]. Using computer-aided design (CAD) software, healthcare professionals could model the anatomical topology from cardiac vasculature to the skeletal system [25, 26]. CT and MRI can provide a 16 bits map with 65.536 shades of gray approximately [27, 28], allowing medical specialists to diagnose and engineers to design models by the identification of the region of interests (ROI) [29]. The volumetric representation of the ROI is obtained by a format called Standard Triangle Language (STL), which is the first step to create the physical model using additive manufacturing techniques (AMT) [30]. Currently, the main AMT includes, but is not limited to fused deposition modeling (FDM), stereo-lithography (SLA), selective laser sintering (SLS), selective laser melting (SLM), electron beam melting (EBM), and direct energy deposition (DED). This section focuses on describing these 3DP principles and its application on the field.

FDM is the most widespread AMT that uses a heated nozzle to melt engineering thermoplastic, such as lactic polyacid (PLA), acrylonitrile butadiene styrene (ABS), and polymethylmethacrylate (PMMA). The extrusion nozzle is built in an XYZ axis Cartesian robot platform to build layer-by-layer 3D parts [31]. FDM demands no special ventilation; however, high room temperature variation can affect the process [31, 32]. FDM was successfully used to fabricate patient-specific implants with varying densities for cranial defects and femur parts [33] and biocompatible nanocomposites for tissue engineering applications [34, 35]. One disadvantage of FDM, since it works with thermoplastics, is that it can only be sterilized using cold solutions [36].

Stereolithography (SLA), also known as photo-solidification or resin printing, creates 3D parts layer-by-layer through photo-polymerization [37]. SLA uses optical light to scan over a reservoir filled with light-curable resin and induces the molecules to link and solidify specific resin surface regions. Printed parts by SLA method are especially good to recreate cavities, such sinuses and neurovascular channels [37]. The disadvantages of the SLA model structure include low mechanical strength and long manufactory time [38, 39].

Selective laser sintering (SLS) is based on the fusion of powder. It employs a high-power laser as a heat source to sinter powder material (usually nylon, polyamide, or metals) to build-up 3D parts layer-by-layer [40]. The powder material diameter should be in the order of 50 *μ*m to improve the model's mechanical properties and topological surface. Unlike SLA, this process is self-supporting, and each layer is deposited over another. SLS shows promising applications for bone tissue engineering [41] and other numerous biomedical applications such as oral, maxillofacial, neurological, and orthopaedics surgery [39, 40, 42]. Selective laser melting (SLM), a subcategory of SLS, is used to fully melt the powder material and bind them in layers, instead of only fusing the metal powder to bond specific regions.

Electron beam melting (EBM) employs an electron beam in a high vacuum chamber at a very high temperature to melt the metal powder and fabricate metal parts layer-by-layer [26, 43, 44]. Typical materials used in the EBM process for surgery applications are commercially pure titanium (CP-Ti), titanium alloys, stainless steel, magnesium alloys, and nickel alloys [37, 43, 45, 46].

Directed energy deposition (DED) concentrated a heat source, such as an electron beam or a laser, to melt in situ delivery of powder to fabricate 3D objects [47]. Beyond manufacturing layer-by-layer 3D objects, DED can also restore existing parts or add material over the currently fabricated structure and perform surface modification [44]. DED processes have a better cooling effect and refabricating capability [48]. As a disadvantage, DED presents low fabrication efficiency compared to EBM and SLM [26].

Finally, SLS, SLM, EBM, and DED are attractive methods to manufacturing porous metallic structures with complex shapes, which are well desired for patient-specific surgical implants to improve bone-in-grown and reduce bone-metal elastic modulus mismatch, thereby allowing for long-term implant stability [26, 49, 50]. However, one disadvantage is the residual stress that may cause interlayer debonding or crack [26]. The most common materials used for this purpose are CP-Ti and titanium alloys [26, 45, 51].

## 3. Surgical Applications

### 3.1. Craniofacial and Nervous System

#### 3.1.1. Head and Neck

**Figure 1:**
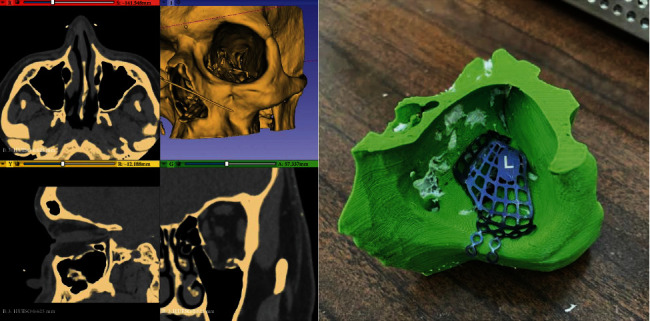
Computerized tomography (CT) of coronal, transverse, and sagittal planes that shows the orbital floor fracture. 3D-printed CT model is shown with the appropriate mesh, and this technique is used to provide a better approach in the treatment of skull fractures. Used with permission from the author Dr. Javier Asensio-Salazar.

3DP offers the possibility to understand complex structures, fractures, and malformations in craniofacial and head and neck surgery. Moreover, the biggest impact of this technology arises when merged with virtual preoperative planning. As a result, surgical CAD/CAM (computer-aided manufacturing) guides and patient-specific implants (PSI) can be created to improve surgical precision and reduce surgical time despite performing increasingly complex reconstructions [52, 53]. Due to its intricate anatomy and important cosmetic function, 3DP is having a relevant impact in this anatomical region (Figure 1). In fact, Pettersson et al. reported in 2019 that the craniomaxillofacial surgery department designed 73.5% of the 3D-printed implants used in Finland per annum [54].

The most common surgical application of this technology in craniomaxillofacial trauma is in orbital floor fracture reconstructions. The goal of this type of surgery is to preserve the shape and volume of the orbit, restore its function, repair any aesthetic impairment, and prevent future sequelae. This procedure involves placing a standard preformed mesh or a PSI. There are two main types of PSI: physically prebent in patient-specific 3D model implants and patient-specific manufactured implants. ORBITA III randomized multicentric clinical trial showed that PSI could restore orbital volume more precisely than standard preformed ones [55]. Further studies comparing those groups of PSI have reported that patient-specific manufactured implants may be superior for orbital reconstruction (orbital volume difference *p* = 0.029) [56]. Hence, improvement in outcomes seems to be related with the degree of customization of the implant.

Furthermore, 3DP proves to be of use in congenital surgery. Le Fort osteotomies and monobloc frontofacial advancement surgery benefit from a combination of virtual planning and surgical guides. Computer-assisted surgery is particularly useful for management of complex cranial malformations such as plagiocephaly, oxycephaly, hypertelorism, Crouzon disease, and Treacher Collins syndrome [57, 58]. Moreover, these types of surgeries can be performed in combination with orthognathic surgery and temporomandibular joint reconstruction. 3DP is having a deep impact in these procedures as orthognathic surgery was at the forefront of virtual planning development, and TMJ reconstruction surgery was an early adopter of custom-made prosthesis [59, 60].

Head and neck surgery also benefit from rapid AM [53]. The most common application is related with bone resection and reconstruction during oncologic surgery. In these cases, fibular flaps can be virtually designed, and guides can be printed to increase precision and reduce surgical time [61, 62]. In addition, dental rehabilitation can be performed simultaneously with dental implants [62]. Furthermore, 3DP can have a special impact in education. Although in its early stages, positive interventions have been published for teaching head and neck anatomy at undergraduate and graduate level [63, 64], thus, increasing anatomical understanding and reducing dependency on cadaveric workshops.

Cost-effectiveness of 3DP in various areas of medicine is yet to be assessed. However, maxillofacial surgery is at the forefront in the validation of this technology under several clinical trials [18]. For instance, Ayoub et al. concluded in a 2014 clinical trial that CAD surgery significantly shortened the time of transplant ischemia and defect reconstruction [65]. Dumas et al. described manufacturing costs for a 3D-printed skull model of $200 (labor cost included) with a turnaround time of 24 hours [66]. These papers advocate for the economic feasibility of 3DP [67].

Future prospects in head and neck reconstructive surgery are paired with tissue engineering and bioprinting. Recent studies have shown how novel biomaterials and polymeric 3DP may aid in the management of congenital pathologies like microtia or alveolar cleft and in acquired tissue defects [68, 69]. In conclusion, rapid AM is reshaping craniofacial and head and neck surgery. It is an efficient solution that improves surgical outcomes and reduces surgical time, especially in pathologies that require fine 3D conformation.

#### 3.1.2. Brain and Spinal Cord

3DP is developing at a rapid pace with countless biomedical applications especially in highly demanding, precise, and technological fields such as neurosurgery. 3DP technologies obviate the need to learn on the patient. This section provides a brief overview of the current state-of-the-art of 3DP applications in neurosurgery focusing on three general areas: (a) creation of 3D-printed patient-specific anatomical and pathological models; (b) creation of 3D-printed neurosurgical instruments, devices, and implants; and (c) creation of 3D bioprinted scaffolding for tissue engineering and research.

The creation of patient-specific models is perhaps the most impactful application of 3DP technologies and has shown to enhance presurgical planning, surgical simulation with recreation of surgical scenarios and complications (e.g., intraoperative dural sinus injury), surgical training, patient education, and interdisciplinary communication (e.g., awake craniotomies and endonasal endoscopic procedures) [70–76].

**Figure 2:**
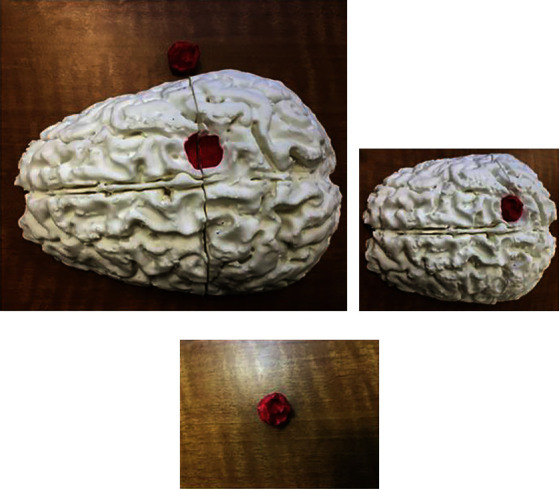
Postprinting result. (a, b) The printed brain can be combined with the tumor print (red) in order to establish the relationships of the adjacent anatomic structures. (c) The tumor can be painted to determine the separation from the brain parenchyma. This is a cost-effective procedure that can help to improve the three-dimensional visualization of the brain tumors to improve the management [71]. Used with permission from Elsevier.

Patients expect utmost proficiency and mastery from their neurosurgeons. Neurosurgical mastery requires comprehensive anatomical knowledge and hours of deliberate practice in the operating room and skill lab. Unfortunately, the case volume and overall surgical exposure during neurosurgical training (for each trainee) have declined as a result of strict duty-hour restrictions and the current global pandemic [77, 78]. Now more than ever, surgical simulation with 3D-printed models plays a pivotal role in neurosurgery training and anatomy teaching [72, 79]. Physical 3D printed models that recreate patient-specific anatomy and pathology can be readily manipulated to help better understand approach-specific and complex pathoanatomical relationships that are otherwise hard to visualize using other traditional means and allow for practicing the different phases of the operation in a safe environment (Figure 2). One area of growing interest is open cerebrovascular neurosurgery—a daunting field that requires immediate action [80] and unique surgical dexterity. Numerous studies have shown the utility of 3D-printed models for presurgical planning, approach selection (e.g., feasibility of using smaller “keyhole” craniotomies), aneurysm clip selection, configuration, and simulation [72, 73, 79, 81].

Other reported applications are brain arteriovenous malformation resection [79, 82] and endovascular techniques [83, 84]. Applications in skull base neurosurgery are 3D-printed models for simulating endoscopic techniques such as endonasal transphenoidal approaches and tumor resections and open techniques such as transtemporal approaches, anterior clinoidectomies, middle, and posterior fossa approaches [72, 79, 85–87]. Applications in hydrocephalus treatment and pediatric neurosurgery are models simulating neuroendoscopic third ventriculostomies and pineal biopsies, external ventriculostomy, and craniosynostosis repair [72, 79]. Noteworthy applications in spine surgery are planning of complex spinal deformity cases, simulators of pedicle screw placement with accurate haptic feedback of cortico-cancellous interface, C2 laminar screw placement, and research [70, 88–90]. Finally, macroscopic and microscopic pathological 3D-printed models can also be used for research by reproducing complex physiology and flow dynamics as in arteriovenous malformation niduses [91] and brain aneurysms even with endothelial lining [74].

3DP allows for rapid and inexpensive prototype manufacturing of surgical instruments (such as microforceps), devices such as patient-specific navigation molds, headrests for frameless gamma knife surgery, synthetic custom-made cranioplasties for covering bony defects, and spinal implants [90, 92, 93]. 3D printed scaffolds can be engineered for biological ingrowth or engrafting with applications in research and transplantation [94]. The complex 3D extracellular microenvironment [95] of human tissues can be replicated using 3DP techniques by providing a physical matrix and incorporating cell-supporting molecules for culturing human and cancer cells, developing tumor models, and manufacturing implantable tissue grafts [96]. The desired external geometry and internal structure of tissue scaffolds are readily controlled.

### 3.2. Cardiovascular System

#### 3.2.1. Cardiothoracic

3DP in cardiothoracic surgery has broad applications [97]. This section is focus on three significant applications: congenital heart defects (CHD) and mitral valve disease, pulmonary interventions, and for educational purposes.

Congenital heart defects (CHD) present with a wide range of complex and unique structures. Traditional imaging methods such as CT, ultrasound, and MRI are not very useful for assessing the unique and usually intricate spatial relationships associated with CHD [98, 99], since its 2-dimension projection differs significantly from the operating room reality. Therefore, 3D-printed models have significant advantages regarding presurgical planning and simulation of cardiac surgeries [100]. These models display with high fidelity the complex anatomical defects of patients with CHD and enable a comprehensive evaluation of the unique spatial relationships that other methods cannot obtain [101].

Several studies show the utility of 3D-printed models in clinical decision-making, interventional planning, facilitating communication between physicians and patients, and enhancing medical education for medical students and surgical residents [98–100, 102]. A recent systematic review assessing the current applications and the accuracy of 3DP for CHD concluded that patient-specific 3D models replicated with high accuracy complex cardiac anatomies demonstrate a substantial value in preoperative planning, surgical simulation, and decision-making and intraoperative orientation [103].

The mitral valve anatomy is difficult to assess, due to its relation to the left ventricular outflow tract (LVOT), its position in the posterior aspect of the heart, and the complex relation between the ventricle, subvalvular apparatus, and the LVOT. Since there are no medical options for the treatment for severe mitral valve regurgitation, its treatment relies on surgical repair or replacement, which faces many challenges [104]. From the steep learning curve, the success of the procedure is based on the surgeon expertise, to the difficult planning before interventions, since it is challenging the interpretation of the valve's anatomy from a 2D or 3D echocardiographic projections [105]. Several authors have used 3DP to simulate different mitral valve pathological process, with rigid plastic and silicone-cast based on the 3D transoesophageal echocardiography. These models allow the physicians for preoperative planning and device testing on minimally invasive valve surgery simulators [106].

Preoperative planning with 3DP is useful for a transcatheter mitral valve replacement (TMVR), an alternative treatment for severe symptomatic mitral valve disease that is not amenable for surgery due to increased intraoperative risk [107]. TMVR has a highly prevalent complication which is LVOT obstruction after device placement (8.2-11.2%). By using a 3D anatomical model, the surgeon can insert a transcatheter valve into the model to simulate and delimitate the neo-LVOT, thus making the necessary modifications to the catheter used to avoid an obstruction after deployment [108].

3DP plays a crucial role in the management of complex respiratory diseases. The high variability in the anatomy of the tracheobronchial tree [109] makes standardized interventional treatments very challenging especially for stent placement.

Tracheobronchial stents are indicated to treat complex central airway obstruction with both intrinsic and extrinsic airway compression [110], to maintain airway patency and provide ventilation to the lung. Due to the broad variability on the diameter of the airway, angles of ramification of the main bronchus, a precise fit can be challenging to achieve, causing complications such as fracture and migration of the stent, formation of granulation tissue, and possible erosion and perforation of the trachea [111]. Patient-specific 3D stents made of different materials (silicone and elastic thermoplastic) that can produce nonstandard geometrical figures could help prevent the later complications associated with unfitted bronchial stents. Additionally, some groups are testing biodegradable stents, as used in cardiology, that could prevent all together the risk of having devices implanted indefinitely [112].

**Figure 3:**
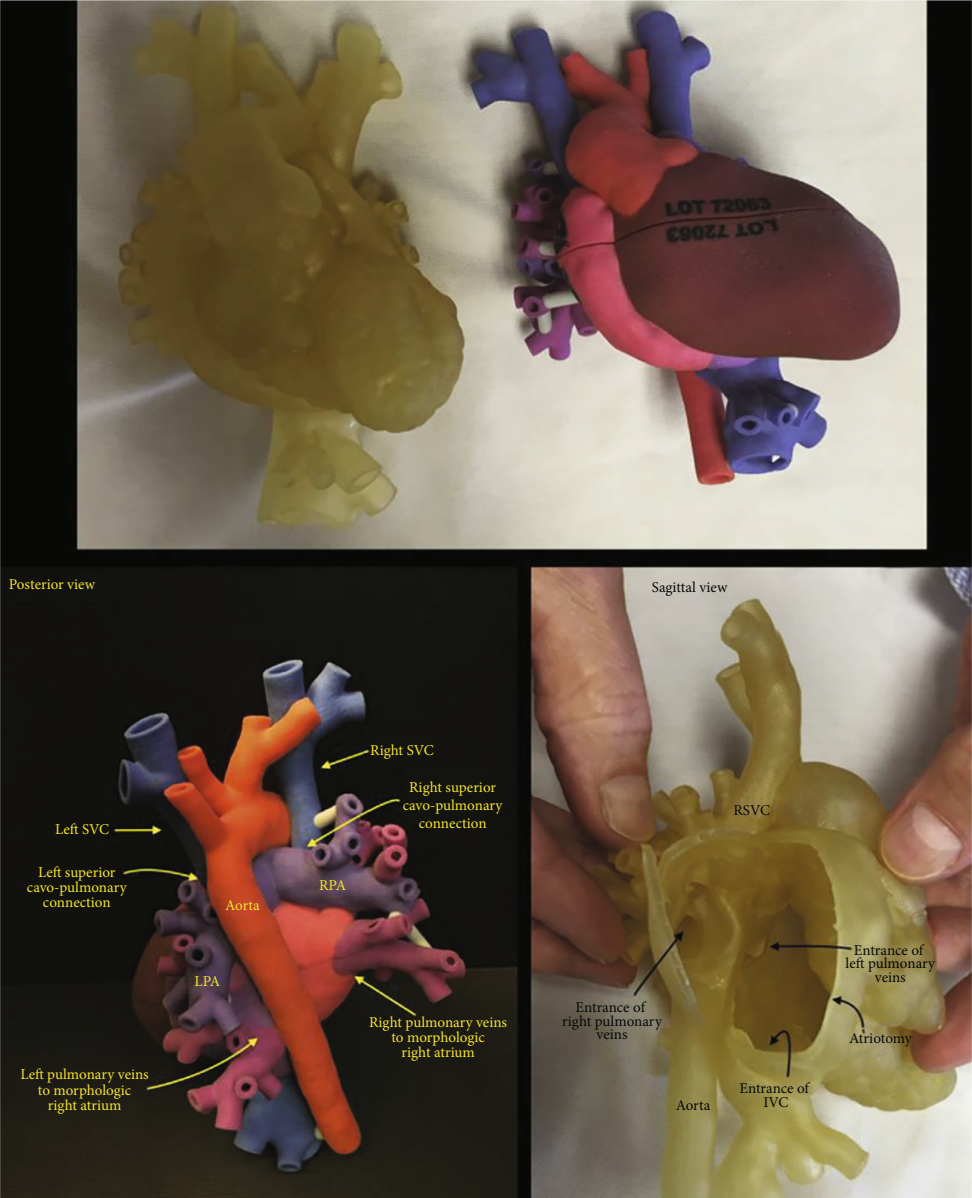
Simulated 3D-printed flexible, intact-heart model used in the surgical planning for complex total cavopulmonary connection. LPA: left pulmonary artery; RPA: right pulmonary artery; RSVC: right superior vena cava; IVC: inferior vena cava; RA: right atrium; Ao: aorta; LA: left atrium; LV: left ventricle; RV: right ventricle; SVC: superior vena cava [117]. Used with permission from Elsevier.

The utility of 3DP, as a didactic tool for educational purposes at every level of training, is extremely promising. 3D teaching engages visual and tactile representations that improve understanding of complex diseases such as CHD, achieving a rapid understanding of anatomical defects hard to depict on a 2D image [101, 113, 114]. Some studies have demonstrated that medical students perform better at identifying cardiac anatomy when using 3D-printed models vs. cadaveric traditional learning [115]. Other studies assessed the efficacy of 3D models of ventricular septal defects as part of a CHD curriculum for medical students showed a statistically significant difference between the experimental and control groups in satisfaction, perceived learning quality, and structural conceptualization [116] (Figure 3).

3DP will play a significant role in the future of care of CHD patients by promoting surgical interventions tailored to the unique CHD anatomy of each patient and creating a dynamic and real-life didactic tool for medical training and communication with patients and caregivers, since teaching patients their conditions with 3D models allows might give them more confidence in explaining their condition, knowledge on their disease, and overall improved satisfaction in the consult [100].

#### 3.2.2. Vascular

Since the first application of 3DP in vascular surgery, in which a life-size replica of the aneurysm was made prior to the endovascular procedure for surgical planning, many studies have been published in the last 20 years [118]. To date, no randomized controlled study on 3D prototyping in vascular surgery is available [119–121], and most of the published studies are descriptive and case series. 3DP in vascular surgery is mainly applied to (a) infrarenal and juxtarenal abdominal aorta aneurysms, (b) thoracic aortic aneurysms, and (c) other approaches to large vessels such as celiac trunk, splenic artery, carotid, subclavian, and femoral arteries, as well as the portal vein.

Most vascular surgeons plan their surgeries using CT and magnetic resonance imaging, in some cases, also Doppler ultrasonography to obtain complementary hemodynamic details [122]. Among the printing techniques, FDM is one of the most popular and least expensive [123]. 3DP is evolving in vascular and endovascular surgery, providing images from large vessels to the smallest ones, allowing total control of the area for planning surgeries. One of the utilities of 3DP is to work aortic fenestrations in complex cases of thoracoabdominal aortic aneurysms, reducing surgical time and achieving an improvement in measurements; achieving to merge an experience of performing surgery while planning, which would reduce interobserver human error during the preoperative phase [124, 125].

**Figure 4:**
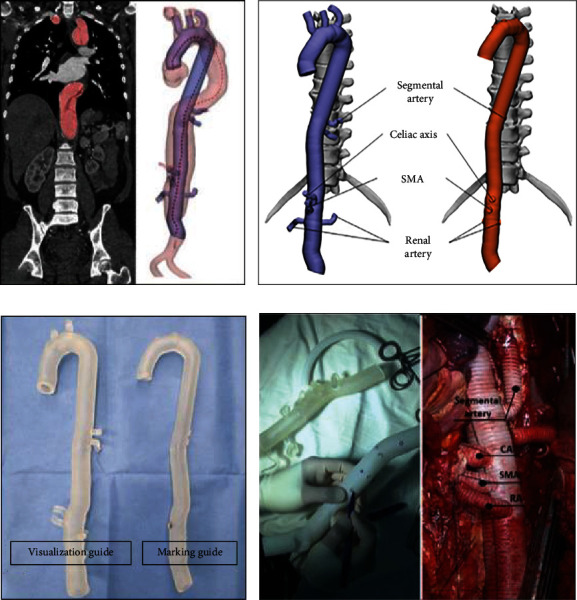
3D modeling processes for 3D aortic model. (a) Individual modeling of the central line of graft using the patient's computed tomography (CT) images. (b) 3D computerized models for the graft guides consisting of the visualization (left) and the marking (right). (c) 3D printed guides. (d) Intraoperative view of the 3D printed guides in the TAAA. SMA: superior mesenteric artery; CA: celiac artery; RA: renal artery; TAAA: thoracoabdominal aortic aneurysm [137]. Used with permission from Elsevier.

This technology allows decreasing the learning curve of vascular surgeons in many centers, reducing fluoroscopy time, and improving decision making in the choice of stents [120]; in complex cases, it manages to discard indications based on experimental models, where the desired results are not achieved, improving decision making in each case ranging from the choice of devices to the angular challenges of the real simulation, but it is the force to which it is subjected that provides great realism in cases of challenging hemodynamic management (Figure 4) [126, 127].

It is important to note that CT measurements for scheduling endovascular procedures are based on the central flow line. However, even modern workstations can not accurately predict the duration of treatment in cases of severe aortoiliac tortuosity [127]. These patients tend to shorten due to the combination of native aortic remodeling, stent conformability, and stiffness of guidewires and delivery systems during endovascular aneurysm repair [128]. Arterial deformations caused by endovascular equipment depend on multiple factors, such as the morphology of the arteries, the state and degree of arterial calcification, and the type of device used [129]. At present, their prediction is mainly based on the surgeon's experience [130].

Attempts to predict the clinical application of 3DP are currently speculative [121, 131]. The additional cost and time required to produce devices with current 3DP still limit their widespread use in hospitals; stereolithographic models can facilitate preoperative planning at least in complex cases. Their limitations are related to the properties of the materials, which do not accurately mimic those of tissues, and their inability to simulate events such as bleeding and other intraoperative complications that surgeons may encounter [132, 133]. However, its great limitation is the manufacturing time, which usually takes 48 hours to have a final model, which significantly delays the corrective power in emergency situations [134].

Some centers already use this technology for the generation of specific models adapted to the patient and training templates for staff [135]. The accuracy of the quality of 3DP adapted to the vascular anatomy of the patient in current comparative studies shows high-quality predictive results (<1 mm difference of the printed vessel wall whether aortic or coronary compared to that predicted in digital imaging and communication in medicine (DICOM) studies), this for FDM and Polyjet technologies [136].

### 3.3. Digestive System

#### 3.3.1. Gastrointestinal

Over time, novel technologies and the introduction of diagnostic imaging have reshaped the practice of surgery. In this context, it allows the creation of graspable, patient-specific, anatomical models generated from medical images. The ability to hold and show a physical object facilitates the understanding of complex anatomical details such as seen in invasive gastrointestinal tumors located, for instance, in the pelvic cavity or in situs inversus [16, 138–140]. Patient counselling, as well as medical education, and surgical training, will definitely benefit from AM. Pietrabissa et al. found that the most common application of 3DP in gastrointestinal surgery was surgical planning, education, training and anatomical comprehension of the disease [141].

**Figure 5:**
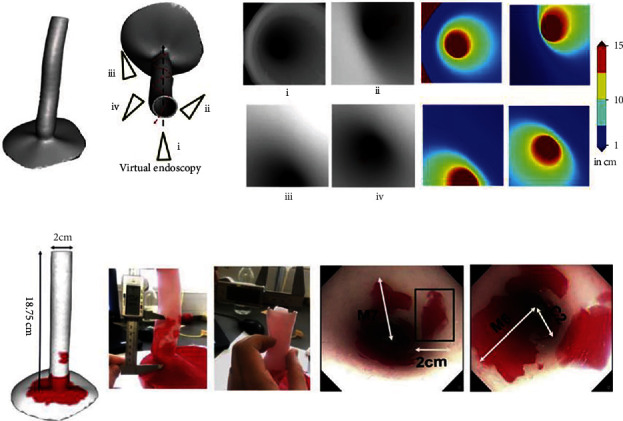
(a) Visualization of 3D depth maps models of the esophagus. Techniques to determine the localization, length, and depth of the Barret's lesions through the endoscopy camera. (b) 3D printed model of the esophagus that shows the measurements for the lesions (C and M) and also the endoscopy video frames are shown [163]. Used with permission from Elsevier.

One of the more challenging procedures in gastrointestinal surgery is esophagectomies and the subsequent reconstruction. To date, the rate of complications is high despite multiple efforts and different strategies [142, 143]. If esophagectomies are performed, this situation has led to the development of alternative treatment technologies like esophageal transplantation. For instance, Takeoka et al. developed a scaffold-free structure with a mixture of cell types using bio-3DP. His team transplanted the structure into the esophagus of murine models with good functional results. The successful outcome was related to the higher content of human bone marrow-derived mesenchymal stem cells [144, 145]. In a similar study, and using tissue-engineered scaffolds, Park et al. demonstrated that those which had 3DP polycaprolactone (PCL) scaffolds presented better muscle regeneration. Also, better epithelialization was observed with polyurethane- (PU-) nanofiber (Nf) scaffolds [146]. In the case of inoperable esophageal tumors, the major treatment of choice in order to alleviate dysphagia is the use of esophageal stents such as self-expandable metallic stent (SEMS) and self-expandable plastic stent (SEPS), however, there is a current development of novel personalized 3D-printed esophageal stents with the goal of improving the symptoms and to provide local anticancer therapy (Figure 5) [147].

In the case of complex anatomy, 3DP is useful for the better visualization and planning of complex surgeries. Some potential examples of the advantageous use of AM technology are in the surgical planning of aortoesophageal fistula repairs and laparoscopic resection of multiple esophageal diverticula [148]. Having a 3D anatomical model resembling the anatomy that will be explored in the OR enables to develop strategies for saving time, with optimum postoperative results.

In terms of medical education and training, multiple institutions like the University of North Carolina and the University of Toronto have acknowledged the need of 3D-printed organs for a surgical simulation curriculum [149, 150]. Some procedures that started to use 3DP for training are in the treatment of pyloric stenosis [151] and dissection of the complex vascular anatomy of the celiac trunk [152]. A recent paper published by Stier et al. showed a novel use of quantitative three-dimensional computed tomography volumetry (3D-CT) of the upper gastrointestinal tract in bariatric surgery, where they found that it facilitates the identification of the postsurgical three-dimensional gastric anatomy and also can be used as an additional diagnostic tool in postbariatric patients with postprocedural complications or prior to revisional procedures [153].

The use of 3DP in colorectal surgery has also impacted the treatment at many levels. For example, the use of individualized models of stomas in order to educate patients and find the right fit of ostomy bags in patients with difficult abdominal wall anatomy [154, 155][17, 18]. With regard to preoperative planning and intraoperative guidance for laparoscopic resection of liver metastases due to colorectal cancer, it is evidenced in the review done by Emile and Wexner that 3D models were supportive, especially in those tumors that were not palpable or recognized by intraoperative ultrasonography, as well as described in by Witowski et al., where obtained that the patients presented a decrease in postoperative complications, being considered a cost-effective technique [154, 156, 157].

One of the most promising aspects of surgical planning and 3DP is pelvic surgery. Hamabe and Ito developed a 3D model of pelvic muscles and neurovascular structures for total mesorectal excision (TME) and lateral pelvic lymph node dissection (LLND). They demonstrated a better anatomical recognition that facilitates the dissection and resection with optimum postoperative results [158]. Also, another novel 3D-printed device was carried out by Rodriguez-García et al. to perform transanal endoscopic surgery without pneumorectum [159]. Chen et al. demonstrated that the preoperative use of 3D models for the resection of right colon tumors resulted on a decrease in operative time, amount of bleeding and greater resection of lymph nodes in comparison with those cases in which these models were not used [160]. Likewise, Sahnan et al. demonstrated that, when 3DP is used for the repair of anal fistula, the results this technology improve the anatomical assessment and its correlation with imaging during surgery, as well as serving as a medium to enhance the education of trainees and a useful adjunct for communication with patients [161, 162].

Like every developing technology, it is still facing several limitations. For instance, the lack of elasticity of some materials precludes the use in certain cases where dissection planning is key. In terms of surgical planning, the high cost of 3DP equipment may limit the use of this technique in settings of limited financial resources; therefore, surgeons may choose to limit such techniques to complex or refractory cases. Future improvements in clinical applicability, in terms of raw materials to print with, speed of printing and reduced costs, are likely to make 3DP more widespread.

#### 3.3.2. Hepatopancreatobiliary

The impact and influence of 3DP in hepatopancreatobiliary surgery have become a relevant and innovative tool for planning and performing complex surgeries, management of malignancies as well as surgical trainee, and patient education [16, 164–168]. The high precision that these models give to the surgeon is useful for the spatial orientation of relevant anatomic structures including portal vessels and hepatic veins, liver segments, biliary system, and pancreatic tumor location thereby enhancing surgical approach [168, 169].

In hepatobiliary surgery, the main application of 3DP is surgical planning, which is approximately 47% [170]. Nevertheless, the usefulness of this technology is not only based on this purpose, in fact, it showed an important value with intraoperative navigation during the surgery, from 80% of success for stent placement and wire manipulation to 100% for needle puncture [19, 171]. Indeed, studying the anatomic structures previous to the surgery and knowing the patient-specific anatomy clearly enhance the procedure [165, 170, 172].

**Figure 6:**
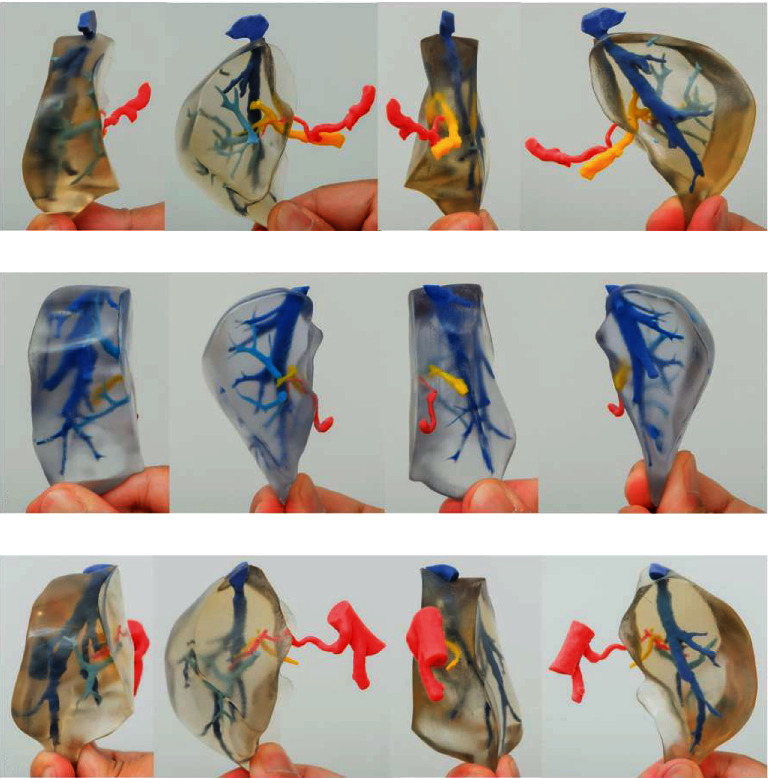
Four aspects of 3D print MSG with portal venous variations. (a)–(c) Shows the different anatomical variations of the portal vein in relation to the medial segmental graft. 3D: three-dimensional; MSG: medial segment graft; P4: portal vein to the medial segment [184]. Used with permission from Elsevier.

The most common surgery for which 3D models were used is the resection of hepatic malignant neoplasms; however, its value in liver transplantation was also outstanding, especially for the use of landmarks and surgical procedure [169, 170, 173]. In spite of the fact that the surgical outcomes were not different among different studies, and most of the studies reported optimal results from the surgical planning point of view (Figure 6) [170]. Randomized controlled trials are necessary for assessing the outcomes.

In biliary surgery, 3D models were mainly used for training purposes of choledochoscopy and ampullectomy, as well as the development of biliary stents [167, 174–176]. However, recently, some studies have shown the inclusion in other biliary pathologies. Zeng et al. reported a great stereoscopic sense in diagnosis and treatment of hilar cholangiocarcinoma; moreover, 3DP models improved the precision of the procedures and better patient outcomes [177]. In addition, Allan et al. found that these models were significantly useful in the treatment of congenital biliary cysts with high accuracy in the replication of anatomical structures, although there were differences in dimensional measurement between the original CT and the 3D model [178]. On the other hand, literature about the use of gallbladder 3D models is still scarce; indeed, it is only known that prototypes from this organ are similar and could satisfy the surgical planning [179].

In the case of pancreatic neoplasms, some case series in pancreatic adenocarcinoma and mucinous neoplasm have published its utility facilitating a more detailed and comprehensive anatomy as well as a clear and direct way for preoperative planning and surgical training [19, 172, 180]. However, there is lack of evidence of the use of this innovative tool in pancreatitis and its complications. Nevertheless, recent studies have extrapolated this 3DP with bioprinting for generation of artificial pancreatic islets, which findings are promising for future treatment for some diseases as type 1 diabetes mellitus [181, 182].

In regard to the patient education, studies stated that 3D-printed hepatobiliary organs reassured the patients giving them approximately a 25% chance of improving in their understanding about anatomy, physiology, tumor characteristics, and the procedure itself, as well as a better sense for the decisions taken for their treatment as well as the surgical risks [166, 172]. Some of the disadvantages of this innovative tool are the time taken for its creation and the cost; indeed, each model has a cost that ranges from 400 USD to 1000 USD, which are factors that have to be taken into account [170]. However, the benefits that this technology gives us are extremely useful for surgeons, residents, and patients, especially in complex cases, hence, as happened with other technological tools, and this one is expected to be widely accessible for several institutions and adopted by more surgeons in their everyday practice in the near future [165, 183].

### 3.4. Genitourinary System

#### 3.4.1. Reproductive

3DP has in recent years become novel and useful tool in the field of obstetrics and gynecology for the preoperative diagnosis and planning of complex female reproductive tract pathologies as well as patient education and the creation of customized devices. 3D-printed models use images generated from CT imaging, ultrasound, or MRI, classically used to evaluate such pathologies, to create anatomic replicas, devices, implants, and surgical instruments customized to individual patients [185, 186].

Currently, the most common application of 3DP in the field of obstetrics and gynecology is the creation of patient-specific 3D-printed anatomic and pathologic models. This has very meaningful uses gynecologic surgery as they allow optimal preoperative surgical planning with improved concordance with intraoperative findings, better surgeon experience with improved conceptualization of the lesion, and ultimately improved patient outcomes [187, 188]. Recent literature has described 3D printed models being used for the successful planning of many surgical procedures, such as for complex female genital tract malformations, cervical cancer [189], multiple uterine myomas [190], endometrial cancer[191], breast cancer tumors [192], and surgical planning of complicated caesarean delivery [193]. Not only can these models be created via noninvasive imaging techniques, they can create accurate life-sized representations of the unique contours of the structures it represents to determine optimal resection margins and approach.

3DP also has a promising role in the evaluation of Mullerian anomalies and rare female genital tract malformations. A case study by Tomlin et al. reported a rare case of unilateral cervical atresia in obstructive hemivagina with ipsilateral renal anomaly (OHVIRA) that was correctly identified preoperatively via 3DP from 3D MRI, but is typically often missed by standardized CT and traditional MRI [186].

3DP can also be applied to the creation of patient-specific medical devices and customization of instruments and tools used in surgery to decrease costs and increase patient satisfaction and comfort [194, 195]. Most medical devices are made in standardized sets of shapes and sizes and often do not provide the best fit for the patient using them. This can result in poor fit and discomfort that can subsequently lead to discontinuation and suboptimal results, thus, necessitating the ability to customize based on the patients' unique anatomic needs. Customized pessary fabrication via a 3D-printed mold, for example, was explored by Barsky et al. to address the common factors limiting efficacy and proper mechanical fit in women with unique anatomic considerations. Likewise, 3D-printed customized vaginal stents and dilators have been successfully created to safely and comfortably fit the pediatric and adolescent population, when none currently exist [196]. Further applications of this extend to the creation of custom 3D printable gynecologic devices that can provide individualized patient-specific tissue stretching to optimize tissue healing and remodeling [197].

**Figure 7:**
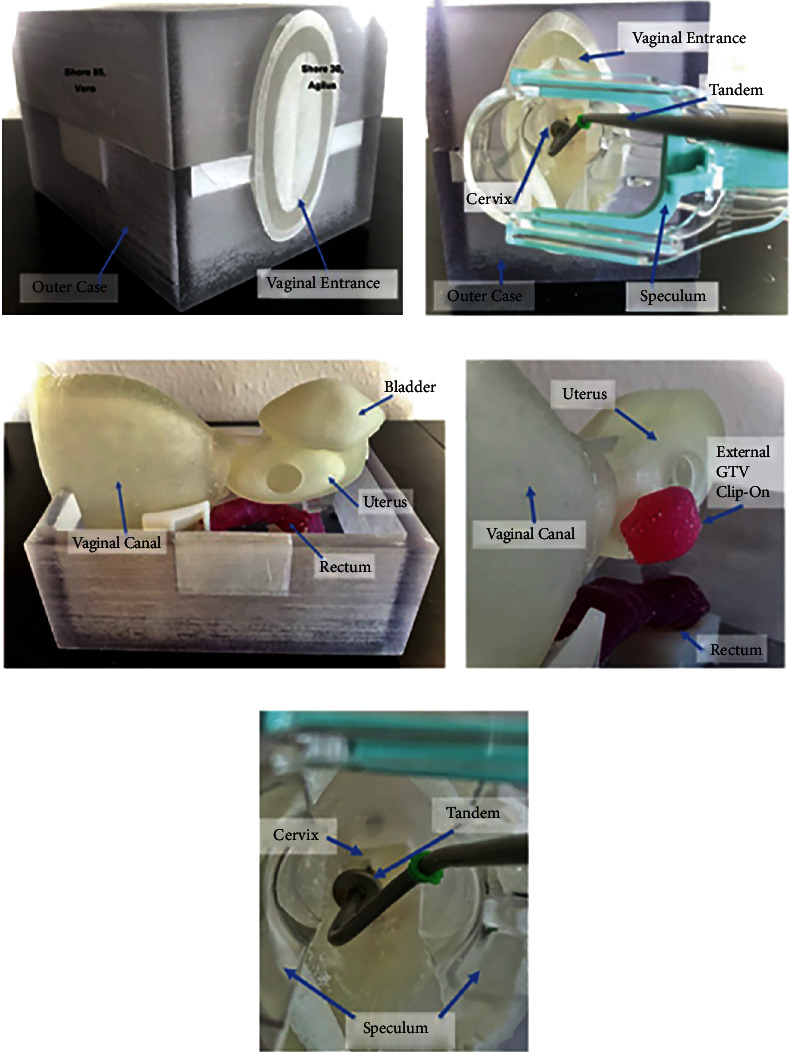
(a) Closed 3D printed model. (b) The speculum entering the vaginal canal. (c) 3D printed intrapelvic organs and its anatomical position. (d) Presence of 3D printed gross tumor attached to the uterine body. (e) Tandem used in brachytherapy procedures is inserted through the speculum and placed inside the cervix and uterine canal. These phantom models help in teaching physicians the process of intracavitary procedures in cervical cancers [201]. Used with permission from Elsevier.

3DP has also allowed for better patient education through the creation of accurate tangible models that allow patients to understand their organ anomalies or the physical context of their tumor in the presurgical discussion and decision-making process [185]. This can help surgeons demonstrate tumor location, volume, and its extent in relation to surrounding structures to help patients come to terms with the feasibility of fertility sparing surgery, such as in the treatment of early stage cervical cancer (Figure 7) [189].

3D-printed pelvic models based on in vivo imaging can also be used for educational purposes among health professionals and sex educators. A novel 3D-printed educational anatomic kit created by Abdulcadir et al. demonstrates models of female and male reproductive anatomy permitting the representation of variations in sex development and morphology, including models of clitorises of women who have undergone female genital mutilation, all of which are based on in vivo imaging [198]. 3DP can also supplement simulation-based medical education, which has a unique place in postgraduate gynecological training. From vaginal repair models allowing residents to train in the repair of injuries resulting from sexual assault [199] to a hemorrhagic cervical cancer model that can be made to bleed, look and feel real [200], these allow residents a low-risk and low-cost opportunity to refine their surgical skills.

#### 3.4.2. Renal

During the last decade, 3DP has gained importance in urology. This multiple dimension technology has shown diverse benefits within the urology field such as improvement of surgical skills, evaluation before hands-on exposure to real scenarios, and improvement of patient education and surgical outcomes. Additionally, when compared with cadaver and animal training, 3DP models have shown superiority due to its lower cost, easier access [202–204], and ability to achieve a realistic surgical experience in a learner-centered environment. This allows repetitive practice, graduated advancement, exposure to multiple clinical scenarios, and errors in a zero-risk platform [205, 206].

Mimicking the characteristics of the human urinary tract using 3DP has been a challenge. Nevertheless, organ models and anatomical phantoms have been created using innovative materials such as wax, hydrogel, agarose gel, polyvinyl alcohol, and silicone. These materials allow recreation of the consistency and anatomy of the genitourinary tract for training, education, and for the development of newer surgical devices [203, 207, 208]. Nowadays, 3DP is mostly used in order to train and plan for partial nephrectomies (PN), percutaneous nephrolithotomy (PCNL), pediatric laparoscopic pyeloplasty, and renal transplantation [204, 205, 209, 210]. Multiple benefits of 3DP in PN include shorter operating and ischemia times [210–212], decreased blood loss, enhanced clamping precision [208, 213], and an improved structural identification [214]; PCNL benefits of preoperative simulation have been observed in mean fluoroscopy times, number of percutaneous access attempts, need for needle repositioning [203], accuracy in stone localization, fragmentation time, and requirement for flexible nephroscopy for stone clearance [209, 215]. As for kidney transplantation, this technology has advanced; in recent years, it has been used as training material in fully immersion simulations with robotic surgery. Studies have found that residents can better understand how to set up and suture the renal artery and vein anastomoses [216], and that after simulation, they can perform the arterial, venous, and the ureterovesical anastomosis within the expected times [217]. Bendre et al. used the Global Evaluative Assessment of Robotic Skills (GEARS) to evaluate residents' performances in robotic-assisted dismembered pyeloplastic before and after training with 3D silicon-based renal models. After training, there was a significant improvement in depth perception, surgical speed, and confidence [218].

Historically, imaging finding is the only available evidence for the patient to understand their diagnosis, possible surgical approach, risks, and possible complications. However, with the development of personalized 3DP, studies have shown that patients describe a better understanding of basic kidney physiology, kidney anatomy, tumor characteristics, better knowledge of the planned surgical procedure, and an overall better comprehension of the disease and the intervention [211, 212, 219–222].

**Figure 8:**
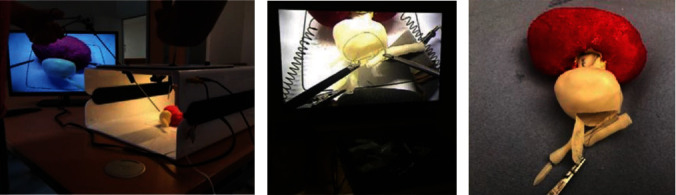
Pyeloplasty is a surgical procedure performed in cases of ureteropelvic junction (UPJ) obstruction. (a)–(c) For surgical training, the 3D-printed models are placed within laparoscopic consoles to recreate and performed the pyeloplasty procedure [226]. Used with permission from Elsevier.

Definitely, the use of this technology appears to be a promising way to improve surgeon's training and enhance the patient's assessment of the procedure. Nevertheless, there are some challenges like the use of more realistic materials, the model's reusability, and the accessibility of this technology in different institutions to overcome the high costs related (Figure 8). Therefore, more surgeons could use 3DP in everyday practice [223, 224]. Many believe that the future of 3DP technology in the urological field consists of building tissue scaffolds with functionality that can grow organs via biofabrication [206, 209, 225]. However, further studies are needed to assess the feasibility of these advances.

### 3.5. Musculoskeletal System

#### 3.5.1. Upper Extremities

**Figure 9:**
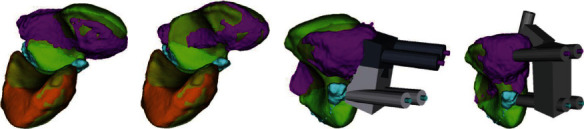
Computer-assisted preoperative planning of a scaphoid fracture. (a, b) Green: scaphoid and lunate bones of the hand. Light blue: proximal scaphoid fragment. Violet: distal scaphoid fragment. (a) Scaphoid fragments before the reduction. (b) Scaphoid fragments after the reduction. (c, d) 3D-printed K-wires are placed in to reduce the two scaphoid fragments in order to have a better sealing of the bone [232]. Used with permission from Elsevier.

As the 3DP revolution unravels in the healthcare field, orthopaedical specific applications are becoming increasingly popular due to the versatility in creating patient-specific components [227]. 3DP enables the next level of personalized patient care by creating custom instruments and hardware [39]. This section will examine the current literature on 3D-printed orthopaedic tools for patient-specific upper extremity malunion correction, primary fracture fixation, surgeon instrumentation and preoperative planning models, and total shoulder prosthesis (Figure 9).

Upper extremity osteotomies are commonly performed to restore anatomical alignment after bony deformity or malunions secondary to trauma [228]. For example, in the pediatric population, 3DP guides for correctional osteotomies for both-bone forearm fractures have been proven advantageous. In a case series out of Osaka, Japan, 20 patients with symptomatic malunited forearm fractures treated with 3D-printed osteotomy guided osteotomies had an improved average forearm arc range of motion and grip strength from 76 to 152 degrees and from 82% to 94%, respectively, compared to the unaffected side [229]. A different case series conducted in Shrined Hospital seven patients with forearm malunion treated with osteotomy with 3D-printed correctional guides had improved forearm supination and pronation by 25 degrees and total rotation greater than 120 degrees [230]. As seen in the aforementioned studies, the implementation of 3D-printed osteotomy guides for forearm fracture malunions can improve range of motion.

The utilization of 3D-printed guides and plates for primary fracture fixation has been explored for scaphoid and distal radius. In the case of scaphoid fractures, historically, high rates avascular necrosis has urged researchers to investigate the benefits of 3DP for achieving anatomical reduction [231]. In a study by Schweizer et al., 22 patients for scaphoid fixation for nonunion with and without 3D-printed patient-specific guides for fracture reduction, authors found that the patient-specific guide group achieved a more accurate fracture reduction with an average residual displacement of 7 degrees versus 26 degrees in the control [232]. In the case of distal radius fractures, 3D-printed patient-specific plates have been found to have higher yield strength than traditional plates, 1043N versus 876N, respectively. The higher yield strength in the 3D-printed plates is theorized to occur due to better mechanical properties of titanium alloy powder or the 3D plate's ability to contour better the patient's bony anatomy allowing for better screw purchase [233].

3D-printed models of patient-specific anatomy can be used to prebend plates. An article out of Chunbgbuk South Korea discusses a technique for using 3D-printed models of fractures to allow for prebending plates for clavicle fractures [234]. These 3D-printed clavicle plates allow for enhanced plate contouring of each patient's unique clavicle geometry, which can have substantial variation based on gender and race. Also, this technique allows for more straightforward fracture reduction, minimizing soft tissue dissection.

The application of 3D-printed patient-specific instrumentation (PSI) for total shoulder arthroplasty has been shown to improve precision and reduce the incidence of component malposition [235–237]. Although many factors influence the functional life of a prosthetic shoulder, suboptimal positioning of the glenoid component has a significant correlation with the risk of implant failure [238]. PSI and custom prosthesis facilitate a reproducible way to improve the accuracy of implant placement, particularly in the setting of severe deformity and bone loss. In a multisurgeon cadaveric study by Throckmorton et al., shoulders with radiographically confirmed osteoarthritis was randomized to PSI or standard instrumentation for anatomic and reverse TSA. Although no difference was found in reverse TSA, in anatomic TSA, PSI improved mean deviation in version from 8 to 5 degrees and inclination from 7 to 3 degrees. Additional clinical outcome studies are needed to define the cost-effectiveness of such technology [239].

#### 3.5.2. Lower Extremities

**Figure 10:**
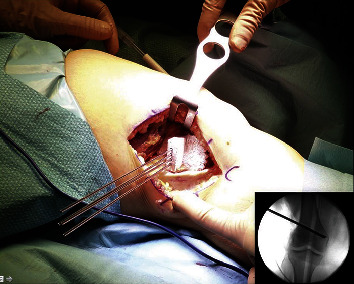
Surgical correction of valgus knees through the use of osteotomy procedure, which consist in the removal or insertion of a wedge of bone near a damaged cartilage in order to provide a well-distributed weight area over the affected knee. Four 3D-printed Kirschner wires are inserted through the guide. Depth and orientation checked under fluoroscopy [249]. Used with permission from Elsevier.

The role of 3DP in lower extremity orthopaedic surgery cannot be understated [240]. Within just a few decades, 3DP has come to play a substantial role in the pre-, intra-, and postoperative stages of treatment in orthopaedics (Figure 10).

3DP provides surgeons with patient-specific anatomic models, enabling extremely precise preoperative planning including optimization of the surgical approach, planning placement of reduction clamps and implants, and the need for additional resources to be used intraoperatively [241–243]. These models accentuate bone defects poorly conceptualized with 2D imaging, allowing surgeons to precisely address the damaged bone and/or cartilage defects and provide the advantage of touch which recalibrates visual perception, enabling a more comprehensive understanding of the clinical problem [242, 244–246]. These models have successfully been used in complex pelvic and acetabular trauma, distal femoral fractures, and distal femoral osteotomies, and have been found to reduce radiation exposure and the risk of iatrogenic neurovascular complications [246–249]. In addition, 3DP simulations and models assist with implant positioning in revision total hip arthroplasty (THA) with complex acetabular defect, with moderate to high accuracy, and satisfied clinical outcome [250].

A large contribution from 3DP has been personalized implants, unique not only to patient's anatomy, but customizable in terms of microstructures (i.e., porosity) and physical properties [251]. 3DP implants are lighter and more comfortable to the patient and can also facilitate minimally invasive surgery [242, 243, 252, 253]. In addition, some implants, like precountered plates, have the added benefit of reducing surgical time and blood loss and improved fit in trauma cases [254]. One of the best-known implants in arthroplasty is the custom triflange which is a patient-specific implant for the treatment of severe bone loss in total hip revisions [246, 255–257]; however, custom knee implants are also used in primary total and partial knee arthroplasty and in patients with previous displaced tibial plateau fractures [256–258].

3DP additionally can create high-resolution bone graft, allowing for exquisite control of porosity and bone interconnectivity, both of which are essential for regeneration and osteointegration [251]. This technology allows, for instance, in situ repair of osteochondral defects through autologous implantation of chondrocytes and bone marrow cells using scaffolds [259]. It also allowed for the incorporation of metallic particles and bone growth proteins into bone graft, which has been shown in animal studies to promote regeneration [260, 261].

Beyond bone graft and personalized implants, preoperative models themselves additionally have the potential to be sterilized and be use intraoperatively, as has been done in complex periacetabular trauma injuries [262, 263]. Other intraoperative 3DP tools have included personalized locking and cutting guides for standard total knee replacements, ACL femoral tunnel guides, and intraoperative models for tibial plateau fractures, distal tibial fractures, talar fractures, and deformity correction [241, 242]. Personalized instruments for pelvic tumor cases have also been found to improve accuracy in simulated surgeries, in addition to reducing simulated operative time [264]. Moreover, 3DP instruments have been found to not only have higher quality than conventional tools but also to be cost-effective [252].

3D-printed models can help assess the degree of anatomy restoration postoperatively [251]. Additionally, customized sockets and prostheses have been made for use after lower limb amputation surgery [265, 266]. 3DP has even been used for rapid production of custom-fit ankle-foot orthoses following subject gait analysis; this custom-fit model was found to at least be comparable to the prefabricated ankle foot orthoses (AFOs) and has been especially helpful in children who are rapidly growing [241, 267].

From preoperative planning to patient-specific models, instruments, and implants; from microscopically engineered bone graft to orthoses, 3DP has quickly been picked up by and applied by orthopaedic surgeons. While the evidence supporting 3DP in orthopaedics is both reflected in recent publication trends and remains promising, there remains room for improvement [240]. Further investigations can focus on maximizing high-value patient care, surgeon education, and patients' functional outcomes related to 3DP technologies. As the field continues to advance—now with 4- and 5D-printing—these different printing modalities have vast potential for simultaneously producing “smarter,” stronger products and enabling excellent patient care [268].

## 4. Exponential Innovations

### 4.1. 3D Printing Integration with Augmented, Mixed, and Virtual Reality

The pressing necessity for the continuous improvement of medical imaging technologies has driven the use of new advanced visualization techniques such as augmented reality (AR), mixed reality (MR), virtual reality (VR), and 3DP in the field of surgery. The goal of these innovative applications is to integrate data found in the medical images into the OR in order to offer patients more precise and personalized treatments [269]. To date, most research has mainly only demonstrated the feasibility of implementation; but the results are quite promising [270].

To visualize or print an anatomical reconstruction from the images, the creation of a 3D-digital model (3DDM) is required [271]. The workflow to build it involves several sequential steps, including (1) 3D data acquisition, (2) anatomical segmentation, (3) conversion DICOM files to a 3D mesh file format, and (4) creation of a computer-aided design (CAD) file [272]. From herein, the resulting model can be uploaded into a virtual space and/or printed as a physical 3D mirror. This process requires dedicated and trained personnel, expensive tools, and time-consuming software [273]. For these reasons, its current implementation in clinical practice remains limited.

**Figure 11:**
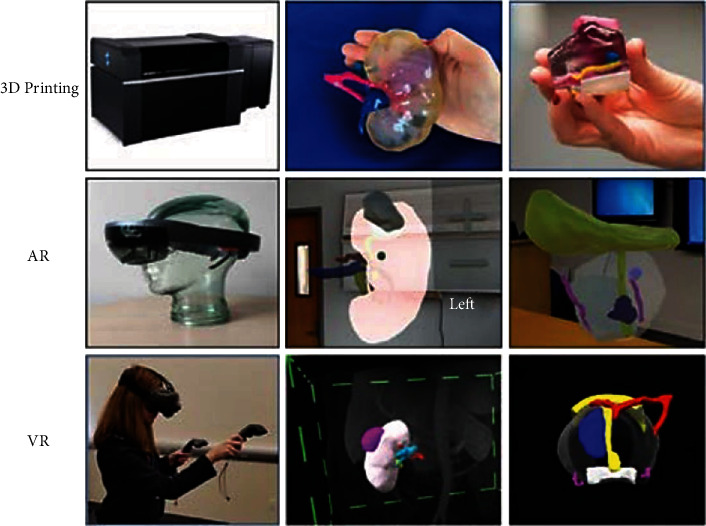
Examples of 3D printing (top), AR (middle), and VR (bottom) technologies. Top row: Stratasys J750 Digital Anatomy Printer; 3D printed kidney tumor model with the kidney in clear, collecting system (semi-transparent), lesion (purple), renal artery (pink), renal vein (light blue), and collecting system (dark blue); 3D printed prostate cancer model with the prostate clear, lesion—blue, neurovascular bundles (yellow), rectal wall (white), bladder neck, and urethra (pink). Middle row: HoloLens-AR headset; AR kidney tumor model shown projected in a room with the kidney (pink), tumor (gray), artery (red), vein (blue), and collecting system (yellow); prostate cancer model shown projected in a room with the prostate (transparent), lesions (blue), neurovascular bundles (purple), bladder neck, and collecting system (yellow). Bottom row: person wearing HTC Vive VR headset; kidney tumor model; prostate cancer model configuration colours as at the middle row picture, also with arterial supply (red) [274]. Used with permission from Elsevier.

In surgery, there are three areas in which the integration of 3DP with AR, MR, and VR is gaining special attention: preoperative planning, intraoperative support, and education training. For example, it has been applied on prostate and kidney cancer, showing improvements in clinical outcomes, surgical planning and intraoperative guidance, and training (Figure 11) [274]. The benefits of these technologies in preoperative planning lie in the increased awareness of anatomical details in complex surgical cases and the ability to identify risks and challenges before surgery, allowing the planning of a safe surgical strategy and potentially avoiding the occurrence of unexpected events [275], a surgical “Mental Map.”

As an intraoperative support tool, 3DDM has also proven their usefulness, reducing the dependence on preoperative interpretation of imaging data and providing an anatomically correct, “twin” model in real time [276]. AR platforms superimpose 3D virtual models on physical objects in real space, allowing simultaneous interaction with both [277]. In this regard, even though, the main limitation remains the lack of accuracy in spatial registration, in Orthopaedic Oncology, it could be beneficial in tumor resection surgeries. Additionally, this technology could be advantageous to guide osteotomy cutting planes [278]. Recently, there have been developments of systems that, from selected anatomical segments in the 3DDM, allow printing and incorporation into the operative field, of a customized patient reference that provides an automatic registration of the image in real space through a smartphone augmented reality application [279].

Today, advanced 3D modeling (3DDM) and visualization technologies have an exciting and most promising range of applications in the fields of simulation, education, and training. The integration of AR, MR, VR, and 3D printing enables efficient training of physicians [280]. Preoperative evaluation and simulation using 3D imaging data allow surgeons to gain valuable experience and preoperatively practice surgical steps in a safe setting [281]. Additionally, these technologies allow real surgeries to be supervised by telepresence [282]. There is no doubt that recent progress in the integration of simulation and virtual modeling into the OR has highlighted the potential benefits of these technologies, however, the concrete clinical impact on operative and postoperative outcomes remains to be defined [283].

### 4.2. 3D Printed Medical Devices and Tools for Space Surgery

Human space activities in recent decades have mostly taken place on the International Space Station (ISS); however, there is growing interest in exploring beyond low Earth orbit (LEO), such as the Moon and Mars. Operational requirements and constraints are closely linked to mission objectives, destination, duration, and necessary resources, influencing the scope of human activity and technology needed to complete missions. Sustaining and supporting the human presence in space require the use of medical devices compatible with the space environment and artificial environments of a spacecraft or planet-based habitat, also considering operational limitations [284].

Preserving human health in space is primarily done through telemedicine-compatible equipment, allowing ground-based experts to be part of examinations. However, the great distances in planetary exploration lead to time delays in communication with medical personnel on Earth, affecting the application of telesurgery in space missions, a technique already shown as a useful tool when there is the need to invasively treat patients who are geographically separated from their physicians [285].

The practice of space medicine also relies on the Crew Health Care System, essential for ensuring crewmember health and safety, including Countermeasures System, Environmental Health System, and Health Maintenance System. Upcoming missions beyond LEO are planned based on the perceived threat level to health or life of an operation and the identified levels of care needed, on which planning of the medical support is based [286]. Surgery in space is thought possible in missions with a level of care four or higher rating, such as in crewed LEO missions and lunar/planetary surface exploration, when lasting more than 30 days. A wide variety of surgical procedures have already been conducted during simulated zero gravity parabolic flights [286]. Nonetheless, there are challenges to performing surgery in a microgravity environment, especially in deep space missions, which includes surgical and anaesthetic procedures and techniques, and ongoing crewmember training to maintain surgical skills throughout a mission [287].

Although space surgery is a high interest area, there is a current lack of validated devices, procedures, and training. Moreover, existing levels of care are built on data from an astronaut population only, not considering potential health issues arising from commercial astronauts or space tourists. The medical history and training of these latter groups are extremely variable; meaning, an equivalent level of care for commercial activity may require surgery as a need/option for shorter missions. Simulated crewed space missions, known as analogue missions, offer a controlled environment for recreating scenarios where surgery may be fundamental, such as medical emergencies [288].

**Figure 12:**
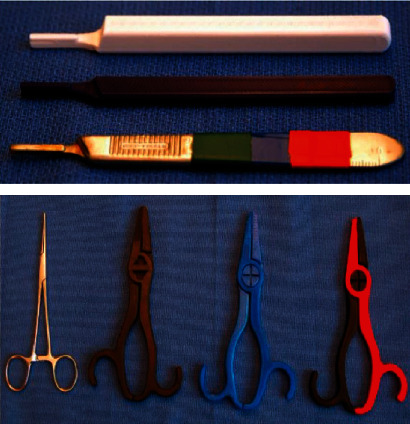
3D printed instruments for space surgery applications [290]. Used with permission from Elsevier.

The possibility of applying space robotic surgery might be impractical, due to the size, weight, and time-sensitive nature of surgical robots [287]. Medical devices, such as blood collectors, are examples of versatile tools used for both clinical research and health monitoring [289], and some can be produced in space using 3DP. Another possibility is to produce medication in space, although little is known regarding their pharmacodynamics and pharmacokinetics in microgravity. More research is also required to identify ways of safely sterilizing and recycling 3D printed medical devices, robotics [6], and tools (Figure 12). Space surgery is still in its infancy; however, it is ripe for innovation and could benefit particularly by combining with the rise and expansion of frontier technologies like artificial intelligence and augmented reality [290].

## 5. Conclusion and Future Insights

3DP is a manufacturing process that has ramped its participation into industry as it offers unique characteristics with the aim to produce objects in a digital fabrication workflow. It has been well-developed in recent years reflected in a variety of surgical specialties, such as head and neck surgery, neurosurgery, general surgery, cardiovascular surgery, urology, gynecology, and orthopaedics. The main impact of this tool is divided into 3 pillars: medical training, surgical planning, and patient education. Obtaining anatomical models of the pathology of interest allows better comprehension and permits an accurate surgical approach. The applications are classified into three different categories: (1) presurgical tool, (2) intrasurgical tool, and (3) implant or replacement. The main manufacturing techniques are fused deposition modeling (FDM), stereo-lithography (SLA), selective laser sintering (SLS), selective laser melting (SLM), electron beam melting (EBM), and direct energy deposition (DED).

The exponential innovations of this technology are having high expectations that will provide a major benefit in the future. In turn, the research and development of this technology have a potential impact with the integration of augmented, mixed, and virtual reality. Besides, other important applications are medical devices and tools for space surgery in order to bring better patient management during spaceflight (diagnosis and treatment). Finally, there is an interesting growing field called “Endoscopic - Intracorporeal 3D Bioprinting,” which consists of creating tissues that help to regenerate damaged organs during a robotic surgery procedure.
